# Using Machine Learning to Predict Obesity Based on Genome-Wide and Epigenome-Wide Gene–Gene and Gene–Diet Interactions

**DOI:** 10.3389/fgene.2021.783845

**Published:** 2022-01-03

**Authors:** Yu-Chi Lee, Jacob J. Christensen, Laurence D. Parnell, Caren E. Smith, Jonathan Shao, Nicola M. McKeown, José M. Ordovás, Chao-Qiang Lai

**Affiliations:** ^1^ USDA ARS, Nutrition and Genomics Laboratory, JM-USDA Human Nutrition Research Center on Aging at Tufts University, Boston, MA, United States; ^2^ Department of Nutrition, Norwegian National Advisory Unit on FH, Oslo University Hospital, University of Oslo, Oslo, Norway; ^3^ Nutrition and Genomics Laboratory, JM-USDA Human Nutrition Research Center on Aging at Tufts University, Boston, MA, United States; ^4^ Statistical and Bioinformatics Group, Northeast Area, USDA ARS, Beltsville, MD, United States; ^5^ Nutritional Epidemiology Laboratory, JM-USDA Human Nutrition Research Center on Aging at Tufts University, Boston, MA, United States; ^6^ Friedman School of Nutrition Science and Policy, Tufts University, Boston, MA, United States; ^7^ CEI UAM + CSIC, IMDEA Food Institute, Madrid, Spain; ^8^ Centro Nacional de Investigaciones Cardiovasculares (CNIC), Madrid, Spain

**Keywords:** obesity, machine learning, genomics, DNA methylation, diet, GxE interaction, precision nutrition

## Abstract

Obesity is associated with many chronic diseases that impair healthy aging and is governed by genetic, epigenetic, and environmental factors and their complex interactions. This study aimed to develop a model that predicts an individual’s risk of obesity by better characterizing these complex relations and interactions focusing on dietary factors. For this purpose, we conducted a combined genome-wide and epigenome-wide scan for body mass index (BMI) and up to three-way interactions among 402,793 single nucleotide polymorphisms (SNPs), 415,202 DNA methylation sites (DMSs), and 397 dietary and lifestyle factors using the generalized multifactor dimensionality reduction (GMDR) method. The training set consisted of 1,573 participants in exam 8 of the Framingham Offspring Study (FOS) cohort. After identifying genetic, epigenetic, and dietary factors that passed statistical significance, we applied machine learning (ML) algorithms to predict participants’ obesity status in the test set, taken as a subset of independent samples (n = 394) from the same cohort. The quality and accuracy of prediction models were evaluated using the area under the receiver operating characteristic curve (ROC-AUC). GMDR identified 213 SNPs, 530 DMSs, and 49 dietary and lifestyle factors as significant predictors of obesity. Comparing several ML algorithms, we found that the stochastic gradient boosting model provided the best prediction accuracy for obesity with an overall accuracy of 70%, with ROC-AUC of 0.72 in test set samples. Top predictors of the best-fit model were 21 SNPs, 230 DMSs in genes such as *CPT1A*, *ABCG1*, *SLC7A11*, *RNF145*, and *SREBF1*, and 26 dietary factors, including processed meat, diet soda, French fries, high-fat dairy, artificial sweeteners, alcohol intake, and specific nutrients and food components, such as calcium and flavonols. In conclusion, we developed an integrated approach with ML to predict obesity using omics and dietary data. This extends our knowledge of the drivers of obesity, which can inform precision nutrition strategies for the prevention and treatment of obesity.

**Clinical Trial Registration:** [www.ClinicalTrials.gov], the Framingham Heart Study (FHS), [NCT00005121].

## Introduction

Overweight and obesity are primary risk factors for many chronic diseases and health conditions, including cardiovascular diseases, type 2 diabetes (T2D), hypertension, and cancers (GBD 2015 Obesity Collaborators, 2017). The prevalence of obesity has increased greatly over the last decades. According to the World Health Organization (WHO), 39% and 13% of the worldwide adult population was overweight and obese, respectively, in 2016 (World Health Organization, 2021).

Up to this point, studies have shown that obesity is determined by genetic, epigenetic, and environmental factors, such as diet and lifestyle, and their complex interactions (Albuquerque et al., 2017). On one hand, candidate gene approaches that consider physiological and molecular development of obesity, genome-wide association studies (GWAS) (Locke et al., 2015), and polygenetic risk scores (PRS) (Belsky et al., 2013) have been utilized to determine the genetic predisposition to obesity. On the other hand, diet and lifestyle behaviors, such as physical activity, are critically modifiable factors in determining obesity (Hruby et al., 2016) and are used to develop prevention and treatment strategies. In addition, much nutrigenetics research has been showing the impact of gene-by-environment (GxE) interaction studies in candidate genes, GWAS-identified genes (Corella et al., 2007; Corella, 2009; Parnell et al., 2014), or PRS (Qi et al., 2012; Casas-Agustench et al., 2014). The overall goals of this research field are to predict obesity with precision, to identify modifiable factors that change the risk of obesity, and finally to develop effective approaches to prevent and treat obesity. Effective prediction tools are needed to attain these goals.

GxE interaction refers to modification by an environmental factor of the effect of a genetic variant on a phenotypic trait. GxE interactions can ameliorate the adverse effects of a risk allele to reduce risk or exacerbate the genotype-phenotype relationship and increase risk (Parnell et al., 2014). Incorporating E factors into genetic and epigenetic studies to explore interactions provides potential advantages, such as reducing missing heritability (Visscher et al., 2008; Manolio et al., 2009). GxE research also has highlighted the individual’s variation in response to interventions by changing environmental factors to prevent or treat obesity. Perhaps, more importantly, examining GxE interactions could support the development of precision medicine. Identifying strategies for modifying E factors that are tailored to an individual’s specific genetic background could enhance the effectiveness of interventions that improve health phenotypes. In addition, epigenomic markers, such as DNA methylation, can be interpreted as footprints of environmental exposures (Kadayifci et al., 2018). We included gene (as genotype)-by-DNA methylation site (DMS) interactions in the present study because this can be considered as another type of GxE interactions on a broader scale.

The evolution of omics technology and data, such as GWAS (Locke et al., 2015) and epigenome-wide association studies (EWAS) (Sayols-Baixeras et al., 2017; Wahl et al., 2017), not only has generated a vast amount of data but also deepened our characterization of complex diseases, including obesity and its related traits. Furthermore, applying machine learning (ML) methods to large- and high-dimensional data provided an opportunity to explore the complex data patterns and structure and to predict disease phenotypes (Degregory et al., 2018; Dogan et al., 2018), and such research is still emerging. Thus, this study aimed to develop an integrated ML approach to incorporate omics data, lifestyle features with consideration of their interactions, i.e., GxG and GxE, to predict any individual’s overweight and obesity status using data collected in exam 8 of the Framingham Heart Study Offspring (FOS) cohort.

## Materials and Methods

### Study Samples: Framingham Offspring Study (FOS) Exam 8 Cohort

The Framingham Heart Study (FHS) has been described at http://www.framinghamheartstudy.org/about/milestones.html. The FHS is a community-based longitudinal study; it recruited participants, who self-identified as having European ancestry, in Framingham, MA, beginning in 1948 (Dawber et al., 1951). In 1971, the FOS then recruited the original FHS participants’ children and spouses (Kannel et al., 1979) and re-interviewed them about every 4–8 years thereafter. In the current study, we utilized data from participants who attended the eighth examination cycle (2005–2008) of the FOS (Generation 2). Participants completed dietary and health assessment questionnaires at that time. These data were obtained from dbGaP (https://dbgap.ncbi.nlm.nih.gov, study accession: phs000007.v25.p9 and phs000007.v28.p10; downloaded on September 27, 2017). The age used was the age of an individual at exam 8.

### Genome-Wide Genotype Data

Genome-wide single nucleotide polymorphism (SNP) genotype and imputed data from FHS were downloaded from dbGaP (accession: phs000342.v18.p11) with initial quality control (QC). In brief, ∼500,000 SNPs were genotyped on the Affymetrix GeneChip® Human Mapping 500K Array (Santa Clara, CA) and filtered at the sample and SNP level. QC steps have been described in detail (Liu et al., 2020). SNP IDs, loci, and allelic information were annotated using the 1,000 Genomes Phase 3 downloaded from dbSNP (downloaded date: April 13, 2018) and human genome build GRCh37/hg19. After these QC steps, 1,967 individuals and 402,793 SNPs remained. Data were processed using PLINK 1.9 (URL: www.cog-genomics.org/plink/1.9/) and 2.0 (URL: www.cog-genomics.org/plink/2.0/) (Chang et al., 2015) and Golden Helix®, and genotypes were coded as 0, 1, or 2. The dosages of imputed SNPs were also categorized as tertile categories and coded as 0, 1, and 2 when used as input data during the feature selection step, i.e., generalized multifactor dimensionality reduction (GMDR). For ML model training and testing, we used original values.

### Genome-Wide DNA Methylation Data

Genome-wide DNA methylation was profiled using Illumina Infinium® HumanMethylation450 BeadChip (San Diego, CA) in whole blood DNA. DNA methylation data were downloaded from dbGaP (accession: phs000724.v9.p13). Raw IDAT files were processed for QC as described (Lai et al., 2018). A β score (proportion of the total methylation-specific signal) was used to measure the methylation signal at each methylation site, and the detection *p*-value was the probability that the total intensity for a given probe fell within the background signal intensity. We excluded any CpG probe with a detection *p*-value > 0.01 and missing sample percentage >1.5% or >10% of samples lacking sufficient intensity. We adjusted batch effects across samples and normalized the β scores using the ComBat function in the ChAMP package in *R* (Morris et al., 2014). To account for the heterogeneity of different cell types across samples, β scores of all filtered autosomal CpG sites were used to calculate principal components, using the prcomp function in R (v12.12.1), and the first five principal components were used in all subsequent analyses. This method was used and is similar to a previous study (Irvin et al., 2014). After these QC steps, 1,967 individuals and 415,202 DMSs remained. The normalized β scores of all DMSs were categorized as tertile categories and coded as 0 (lowest), 1, and 2 (highest) when used as input data during the feature reduction step, i.e., GMDR. For ML model training and testing, we used original values. The annotation was based on human genome build GRCh37/hg19.

### BMI and Categorization of Weight Status

We used body mass index (BMI) to classify overweight and obesity in adults. It is defined as a person’s weight in kilograms divided by the square of the height in meters (kg/m^2^). BMI ≥25 kg/m^2^ was defined and coded as overweight or obesity (*n* = 1,403; 71% of *n* = 1,967) and BMI ≥30 kg/m^2^ as obesity (*n* = 591; 30% of *n* = 1,967).

### Dietary and Other Lifestyle Factors Measurement

Usual dietary intake for the previous year was assessed among 2,245 adult men and women in the FOS. Foods and nutrients were derived from the 126-item modified Willett semi-quantitative food frequency questionnaire (FFQ) at exam 8 of the FOS (Dawber et al., 1951; Rimm et al., 1992; Feskanich et al., 1993). The FFQ allowed participants to name ≤4 extra food items that were essential parts of their diets but were not offered among the 126 items. Energy intake was considered implausible and excluded if a participant reported energy intake was <2.51 MJ/day (600 kcal/day) for men and women or >16.74 MJ/day (4,000 kcal/day) for women and >17.57 MJ/day (4,200 kcal/day) for men or if >12 food items were left blank, consistent with the criteria as previously published in the FHS. The energy composition for macronutrients (% from total energy intake) was calculated, and the food items were summarized into 31 food groups. Three diet quality indices were calculated to capture dietary patterns: (1) the Alternate Healthy Eating Index (AHEI) score identified by factor analysis, (2) the Mediterranean diet score (MDS), and (3) the Dietary Approaches to Stop Hypertension (DASH) diet score. All lifestyle factors, such as alcohol drinking, smoking, and physical activity [through a standard exercise questionnaire (Kannel and Sorlie, 1979)], were available on individuals at exam 8 of the FOS. A total of 397 dietary and lifestyle variables were converted into tertile categories and coded as 0 (lowest), 1, and 2 (highest) as input data during the feature reduction step, i.e., GMDR. For ML model training and testing, we used original values.

### Machine Learning

**FIGURE 1:**
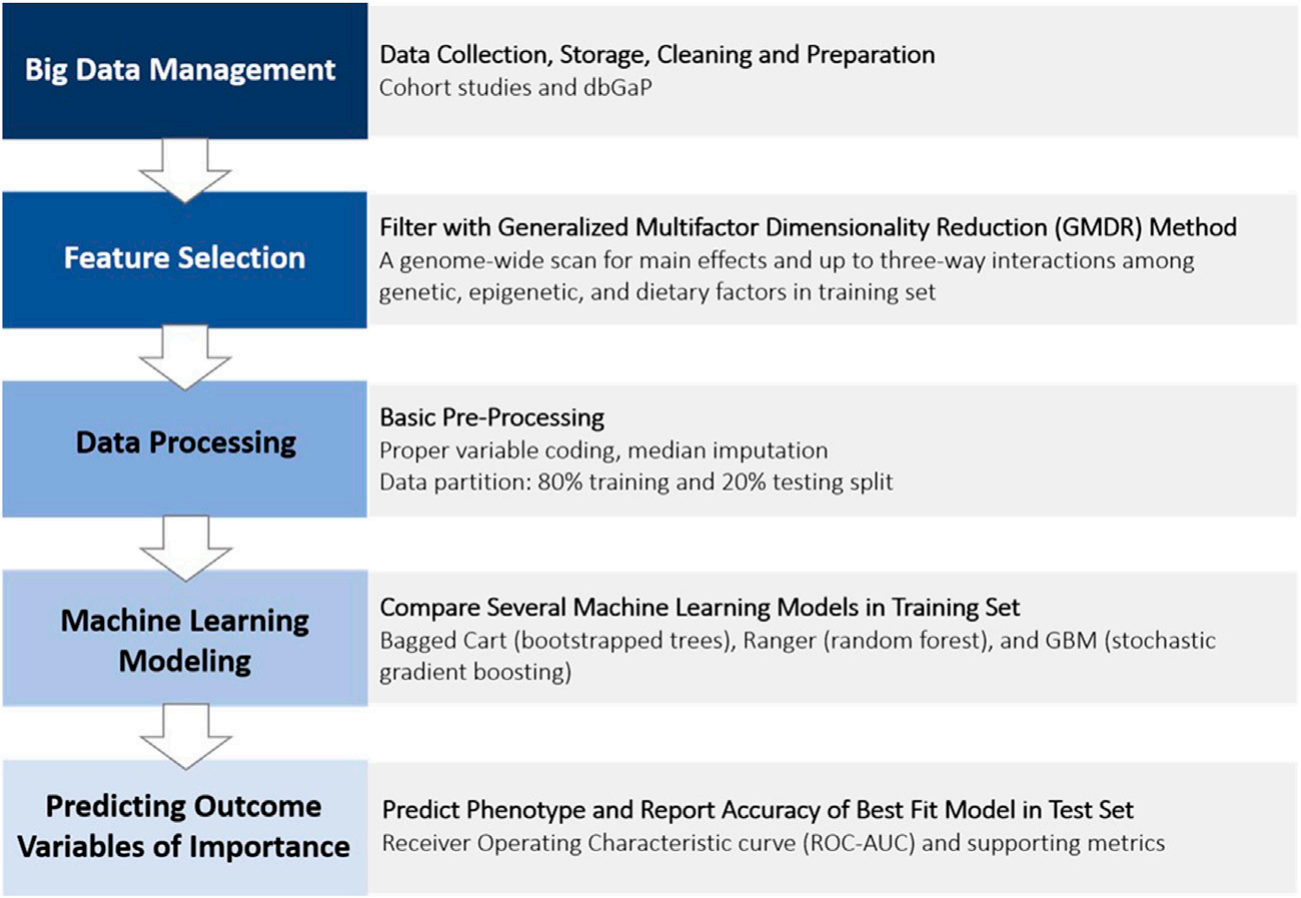
Phenotype prediction data analysis procedure (pipeline).

We used supervised binary classification ML models to predict an outcome variable (e.g., overweight or obese yes or no; obese yes or no). The overall flowchart of ML procedures applied in the present study is illustrated in Figure 1.

#### Training and Testing Data Sets

We derived a final analytic data set of 1,967 Caucasian participants (45% women), aged 40–92 years, who participated in the eighth examination visit of the FOS and had complete data for related demographic, anthropometric, clinical, genetic, epigenetic, dietary, and other lifestyle [alcohol, smoking, and physical activity (Kiely et al., 1994)] variables and covariates. Missing values for some variables were filled using median imputation. Samples were split into a training set (80%) and a test set (20%) by applying systematic random sampling. The PROC SURVEYSELECT procedure and METHOD = SYS (SAS 9.4 for Windows, SAS Institute Inc., Cary, NC, USA) were used to control values of BMI and age within the sex ratio, T2D, and use of lipid-lowering medication. The demographics of individuals included in the training and testing data sets in this study are summarized in Table 1.

**TABLE 1:** General characteristics of the FOS.

FOS	Training set	Testing set
N	1,573	394
Men/women, n (% in women)	700/873 (55.5%)	178/216 (54.8%)
Age, y	66.3 ± 8.9	66.5 ± 8.7
BMI, kg/m^2^	28.1 ± 5.3	28.0 ± 5.2
Overweight and obesity, n (%)	1,122 (71.3%)	281 (71.3%)
Obesity, n (%)	473 (30.1%)	118 (30.0%)
Smoker, n (%)	115 (7.3%)	26 (6.6%)
Drinker, n (%)	1,205 (76.6%)	321 (81.5%)
Type 2 diabetes, n (%)	210 (13.4%)	53 (13.5%)
Hypertension, n (%)	858 (54.5%)	221 (56.1%)
Type 2 diabetes medication, n (%)	160 (10.2%)	39 (9.9%)
Hypertension medication, n (%)	756 (48.1%)	196 (49.7%)
Lipid-lowering medication, n (%)	682 (43.4%)	171 (43.4%)
Total energy intake, kcal/d	1,873 ± 629	1,875 ± 636
Physical activity score	37.7 ± 6.4	37.6 ± 5.8

All continuous variables were presented as mean ± SD.

#### Feature Selection Using the Generalized Multifactor Dimensionality Reduction (GMDR) Method

The GMDR method (GMDR software, Windows version) (Xu et al., 2016; Luo et al., 2017) was applied to the training data set to perform a genome-wide and epigenome-wide scan to detect main effects and three-way GxG and GxE interactions for determining BMI. The GMDR training stage searched attribute combinations with the highest training accuracies. Furthermore, this training performed permutation tests for selected attribute combinations and calculated *p* values based on testing accuracies. This method was implemented to reduce high-dimensional features for subsequent ML steps (10-fold cross-validation (CV), n < 1,000, permutation testing *p* < 0.001). Genotype, DNA methylation, and dietary and other lifestyle data were coded as 0, 1, and 2 as discrete input features to predict the BMI (as a continuous variable) using age, sex, and the first five principal components for DNA methylation as covariates. We ran this procedure five times and collected the union of selected features for the following ML steps.

#### Phenotype Prediction Using Machine Learning Methods

Three sampling-based supervised ML classification algorithms were used to evaluate performance in classifying overweight and obesity: boot-strapped trees (treebag), random forest (ranger), and stochastic gradient boosting machines (gbm). These algorithms were used to generalize the relationship between input features and the labeled examples (output) from the training data and to apply this learning to the prediction of class labels of unseen samples in the test set.

Using the same training data set, we built, tuned, and compared the following models using the caret package and other required packages in RStudio (version 1.3). Caret-automated parameter tuning was used for selecting hyperparameters to establish for each classifier, and a grid of tuning parameters was defined using the expand.grid function. For ranger, mtry, min.node.size, and splitrule were used to set tuning parameters in an optimal range; for gbm, n.trees, interaction.depth, shrinkage, and n.minobsinode were set to search for the best model in the training set. Under-sampling, over-sampling, or synthetic minority oversampling technique (SMOTE) sampling methods were also introduced to address class imbalance. Five repeats of 10-fold CV were set for building the model.

The best predictive models from each algorithm were assessed using the area under the curve of the receiver operating characteristic curve (ROC-AUC). We compared different learning algorithms by using the resamples function using the training data set. We then applied the best model to predict the binary overweight or obesity status in the test data set. The confusion matrix was used to present the overall accuracy, sensitivity, and specificity observed in the testing set samples, which then evaluates the performance of each prediction model. Accuracy is the total proportion of correct predictions of all the predicted data. Sensitivity is the proportion of real positives that are predicted as positives; specificity is the proportion of real negatives that are predicted as negatives. The sensitivity was plotted against 1-specificity to generate the ROC curve.

### Network and Pathway Enrichment Analysis

To identify the enriched pathways of nearby genes of selected SNPs and DMSs, the web-based protein association database STRING (version 11.5) (Szklarczyk et al., 2021) was used to explore possible functionalities of the GMDR-selected features. This tool includes Gene Ontology (GO) enrichment and Kyoto Encyclopedia of Genes and Genomes (KEGG) pathway analyses.

## Results

### Cohort Characteristics

Overall, 71.3% of the FOS study participants were either overweight or obese; 30% of study participants were obese in both training (*n* = 1,573) and test sets (*n* = 394) (Table 1). The prevalence of obesity-related phenotypes was matched between the two data sets (Table 1). For environmental factors, there were no statistically significant differences in the total energy intake and physical activity score (Table 1).

### Features Selected Using the GMDR Method

We used the GMDR method in the training set to conduct a combined genome-wide and epigenome-wide scan for main effects and up to three-way interactions (∼5.5 × 10^17^ combinations) among all pre-filtered 402,793 SNPs, 415,202 DMSs, and 397 dietary and lifestyle factors. The GMDR method identified 213 SNPs, 530 DMSs, and 49 dietary and lifestyle factors that were significant predictors of obesity (permutation testing *p* < 0.001). The complete list of selected features is presented in Supplementary Table S1.

Among 213 GMDR-selected SNP features, there were 131 independent clumped loci based on the PLINK 1.9 clumping function using the greedy algorithm for clumping with linkage disequilibrium (LD) (r^2^ < 0.5) and physical distance (>250 kb). A total of 45 independent SNPs located in or near genes, such as *STXBP6*, *BBX*, *PLXDC2*, *PCDH15*, *TPH2*, *PCDH15*, *CALN1*, *FGF14*, *LRRN1*, *ACTBP2*, *RBMXP1*, and *ZNF32*, were previously reported to be associated with several obesity-related phenotypes and anthropometrics (Locke et al., 2015; Buniello et al., 2019). Among 533 GMDR-selected DMS features, 520 were considered independent signals based on the physical distance and signal correlation. A total of 60 DMS features were found to be associated with BMI and obesity-related phenotypes in other studies (Supplementary Table S1) (Battram et al., 2021). Some DMSs were in proximity to genes, such as *CPT1A*, *ABCG1*, *SLC7A11*, *RNF145*, and *SREBF1*, (Mendelson et al., 2017; Wahl et al., 2017; Dhana et al., 2018; Lai et al., 2020) reported to be related to metabolic phenotypes.

When using a combined gene list of GMDR-selected SNPs and DMSs for predicting BMI to analyze pathway and network enrichments, protein-protein interaction enrichment was significant (*p* = 2.65 × 10^–5^; *p* = 0.00118 when using the top gene list from features of the best-performing model) based on the STRING database (Supplementary Figure S1). Significant KEGG pathways, GO terms, and annotated keywords (UniPort) included Ras signaling pathways, Rap1 signaling pathways, and alternative splicing (Benjamini–Hochberg-adjusted *p* < 0.05). Analyses of the data also showed associations between selected genes with the blood lipid and glucose metabolism that were identified in previous studies. This is entirely plausible because obesity very often coexists with dysregulation of blood lipids and glucose.

### Overweight and Obesity Prediction Using Different Machine Learning Algorithms/Classifiers

**FIGURE 2:**
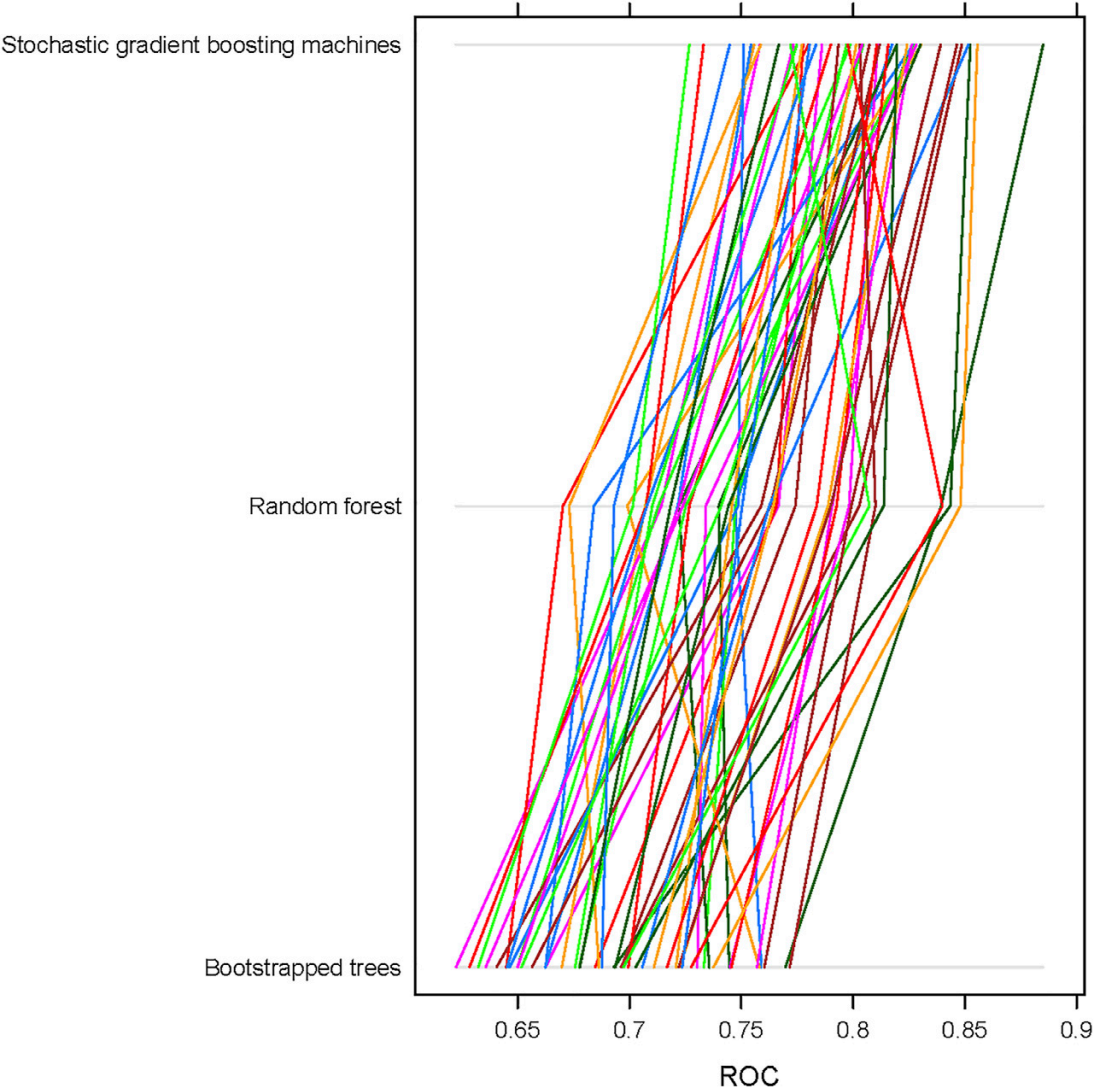
Receiver operating characteristic (ROC) curves and their corresponding AUC values for different machine learning algorithms using 50 sample model objects for obesity status in the training data set of the FOS (*n* = 1,573). All models were based on continuous input variables and under-sampling approach.

We used GMDR-selected features to build ML classification models using three different algorithms: boot-strapped trees (treebag), random forest (ranger), and stochastic gradient boosting machines (gbm). After obtaining the best model in each algorithm, we recorded 50 model objects and compared the performance of these three algorithms using ROC, sensitivity, and specificity. Overall, the stochastic gradient boosting machines (gbm) repeatedly showed the best performance for both overweight + obesity and obesity outcomes no matter which approaches were used to deal with class imbalance. In general, the mean ROC value for stochastic gradient boosting machines (gbm) was ∼0.8 compared to ∼0.75 for random forest (ranger) and ∼0.70 for boot-strapped trees (treebag) in the training set. Figure 2 shows the differences in the distribution of performance of 50 models among ML algorithms/classifiers when applying under-sampling for the obesity status.

**FIGURE 3:**
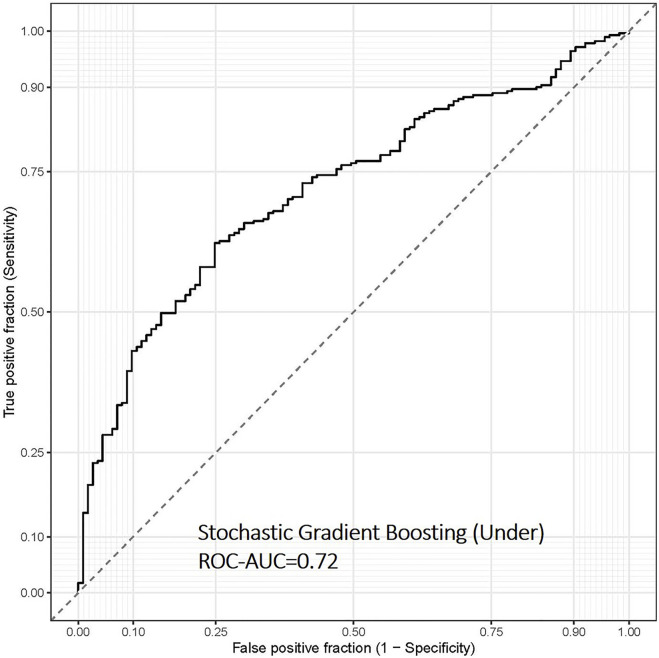
Receiver operating characteristic (ROC) curve of the overweight and obesity prediction model using stochastic gradient boosting machine learning algorithms in the test data set of the FOS (*n* = 394). This model was based on continuous input variables and under-sampling approach.

Finally, we evaluated the overweight and obesity prediction models constructed using various machine learning algorithms in the test set using ROC-AUC, accuracy and sensitivity, and specificity. The stochastic gradient boosting machines (gbm) remained the best model to predict overweight and obesity status in the separate test data set, with ROC-AUC and accuracy values of 0.72 and 0.67, respectively (Table 2 and Figure 3). Depending on different sampling methods used to address class imbalance, the overall accuracy of all models was ∼70%.

**TABLE 2:** Performance metrics of overweight and obesity prediction models constructed using various machine learning algorithms in the test data set of the FOS.

Model/algorithm	ROC-AUC	Sensitivity	Specificity	Accuracy
Overweight and obesity				
Boot-strapped trees (treebag)	0.65	0.64	0.62	0.63
Random forest (ranger)	0.68	0.63	0.64	0.63
Stochastic gradient boosting machines (gbm)	**0.72**	**0.65**	**0.71**	**0.67**
Obesity				
Boot-strapped trees (treebag)	0.65	0.63	0.62	0.62
Random forest (ranger)	0.66	0.53	0.65	0.61
Stochastic gradient boosting machines (gbm)	0.67	0.51	0.68	0.63

All models were based on continuous input variables and under-sampling approach.

The best metrics in each column are shown in bold.

### Important Ranking and Annotation of Top Predictors of the Best-Performing Model

Top predictors of the best-fit model included both genetic and diet-related factors. Compared to SNPs, DMS features predominantly contributed to the best-performing model. In this example, 16 DMSs in genes, such as *CPT1A* (Mendelson et al., 2017; Wahl et al., 2017; Dhana et al., 2018), *ABCG1* (Mendelson et al., 2017; Wahl et al., 2017; Dhana et al., 2018), *SLC7A11* (Mendelson et al., 2017; Wahl et al., 2017), *RNF145* (Mendelson et al., 2017; Wahl et al., 2017), and *SREBF1* (Mendelson et al., 2017; Wahl et al., 2017; Dhana et al., 2018) were reported to be associated with obesity-related phenotypes. Important diet-related factors were processed meat, diet soda, French fries (potato), high-fat dairy, artificial sweeteners, alcohol intake, and specific nutrients and food components, such as calcium and flavonols. We present the top 50 predictors for determining the overweight and obesity status in the test set using the best model of the stochastic gradient boosting machines algorithm (Table 3). In the presence of individual foods and nutrients, dietary pattern variables did not emerge on top.

**TABLE 3:** Top 50 predictive features of the best-performing model for predicting overweight/obesity status in the FOS.

Importance	Feature	Chr	Position	Gene
**100.00**	**Food group—processed meat servings**			
69.43	cg06690548	4	139162808	*SLC7A11*
**52.17**	**cg17061862**	**11**	**9590431**	**NA**
40.14	cg15754660	7	34699393	*NPSR1*
**40.03**	**cg00574958**	**11**	**68607622**	***CPT1A***
36.52	Nutrient value—calcium			
**35.51**	**cg27243685**	**21**	**43642366**	***ABCG1***
33.83	cg06560379	6	44231305	*NFKBIE*
**32.88**	**Food group—diet soda servings**			
28.66	cg11024682	17	17730094	*SREBF1*
**28.62**	**Nutrient value—proanthocyanidin, monomers USDA, 2007**			
28.49	cg05201185	6	30459139	*HLA-E*
**28.19**	**Physical activity**			
28.00	cg17501210	6	166970252	*RPS6KA2*
**25.80**	**cg26403843**	**5**	**158634085**	***RNF145***
25.37	Food—low-calorie Cola, no caffeine			
**25.24**	**cg11998932**	**7**	**3901843**	***SDK1***
24.77	cg26278103	7	124404244	*GPR37*
**24.76**	**cg08677140**	**6**	**30582241**	***PPP1R10***
23.40	Food group—high-fat dairy servings			
**22.75**	**cg01881899**	**21**	**43652704**	***ABCG1***
22.34	rs1740322			
**22.20**	**cg26376241**	**2**	**65594021**	***SPRED2***
21.62	rs4974985	4	38961449	*TMEM156*
**20.35**	**cg03572859**	**8**	**22409634**	***SORBS3***
18.97	cg00174508	12	107774298	*BTBD11*
**18.86**	**cg06500161**	**21**	**43656587**	***ABCG1***
18.02	cg14476101	1	120255992	*PHGDH*
**17.88**	**cg18222913**	**12**	**128846838**	***TMEM132C***
17.22	cg16341269	6	150213172	*RAET1E*
**16.63**	**Nutrient value—proanthocyanidin, dimers USDA, 2007**			
16.58	Sex			
**16.02**	**cg06460869**	**10**	**17270094**	***VIM***
15.75	cg22650271	22	39760165	*SYNGR1*
**15.30**	**cg10426084**	**17**	**1640472**	***WDR81***
15.21	cg08766211	15	79118175	NA
**15.13**	**Nutrient value—isorhamnetin, flavonol USDA, 2003**			
14.77	cg11963676	1	76540110	*ST6GALNAC3*
**13.61**	**cg19978312**	**5**	**179634688**	***RASGEF1C***
13.58	cg04582365	10	59155846	NA
**13.52**	**Nutrient value—epicatechin, flavan-3-ol USDA, 2003**			
13.24	cg07052041	10	135092104	NA
**12.92**	**cg17901584**	**1**	**55353706**	***DHCR24***
12.67	cg18034719	5	176860863	*GRK6*
**12.51**	**Food—French fries**			
12.44	cg15448990	4	88411497	*SPARCL1*
**12.30**	**cg02508743**	**8**	**56903623**	***LYN***
12.29	cg26722769	4	170328730	*NEK1*
**12.27**	**cg25999015**	**19**	**44037866**	***ZNF575***
11.86	cg00945735	7	41982767	NA

### Prediction Using Simulated Data

We further created simulated individual data with different levels of top dietary predictors to observe whether the prediction changes the status of overweight and obesity and at what level of critical predictors switches the prediction class. By changing five key dietary factors individually, we observed 1.5–19.6% of subjects showing responses in changing obesity risk (Table 4). Processed meat showed the greatest response and followed by high-fat dairy and calcium intake. Overall, about 21.5% of subjects showed responses to at least one dietary change based on simulation.

**TABLE 4:** Predicted responses in overweight and obesity status of subjects with simulated dietary feature changes in the test data set of the FOS (*n* = 260).

	Original status		
Modifying feature	Overweight or obese	Not overweight or obese	Total
Food group—processed meat servings	28 (10.8%)	23 (8.8%)	51 (19.6%)
Food group—high-fat dairy servings	15 (5.8%)	3 (1.2%)	18 (6.9%)
Food—French fries	0	4 (1.5%)	4 (1.5%)
Nutrient value—calcium	8 (3.1%)	6 (2.3%)	14 (5.4%)
Nutrient value—animal Fat	5 (1.9%)	1 (0.4%)	6 (2.3%)

## Discussion

We present an ML-based predictive method using genome-wide SNPs, DMSs, and dietary information including up to three-way interactions among these elements to predict obesity. Among ML algorithms, the stochastic gradient boosting model provided the best prediction accuracy for obesity in the training set and overall accuracy of 70% and ROC-AUC of 0.72 in the test set. In each model, predictors of overweight and obesity were identified.

To our knowledge, this is the first study to predict obesity using ML approaches that integrate omics and dietary information in the field of nutrigenetics. While previous studies have used genomics and/or epigenomics to predict obesity or other diseases (Dogan et al., 2018; Cho et al., 2021), we further integrated lifestyle data with genomic and DNA methylation epigenomic data and considered their interactions by applying the GMDR method. By identifying the predictors, our results extend our knowledge about the etiology of obesity. More importantly, selected lifestyle features can inform precision nutrition strategies for the prevention and treatment of obesity by offering options for lifestyle improvement that can be tailored to the individual. We illustrated this concept using simulated data (Table 4). Our results suggested that individuals would respond to different treatment approaches depending on the individuals’ genetic and epigenetic background. With this in mind, it is conceivable that by modifying these top potential “obesogenic” predictors tailored to the individual’s genome and epigenome, the risk of obesity can be reduced at the level of the individual.

Genome-wide association and gene–lifestyle interaction studies made it clear that genetic factors predispose individuals to obesity, but such susceptibility can be attenuated by healthy lifestyle choices. In the current study, we have identified diverse diet-related factors that contribute to predicting the overweight or obesity status. Some factors such as processed meat, high-fat dairy, and diet soda (Mozaffarian et al., 2011) have been investigated for their relationship with obesity; while other factors such as the plant-based compounds flavanols or anthocyanins require further research to define how these factors orchestrate with human genome and epigenome to contribute to obesity. However, due to the larger number of loading features, the nature of complex interactions, and ML approaches used in the present study, further analyses in ML techniques are needed to define the roles of modifiable lifestyle predictors when developing a prevention strategy to mitigate obesity. This type of research will eventually contribute to precision nutrition strategies to maintain healthy weight through controlling diet and lifestyle behaviors.

In this study, individual food items and nutrients appeared to be more important than dietary pattern features. This aligns with the concept of personalized nutrition. The same level of dietary score could be achieved by many ways of dietary intake, and our research suggested paying attention to individual food items or specific nutrients which fit each person’s genetic and epigenetic background. Our data showed that processed meat and animal fat played more important roles than a certain dietary pattern or total fat intake in predicting obesity, but which food items to use in recommendations would depend on each individual. Clinical trials are warranted to validate our findings; in other words, to test the complex interactive relationships between genetic background by changing diet (or lifestyle) according to model outputs. The eventual goal of our research is to understand an individual’s susceptibility to obesity and his or her responsiveness to personalized interventions in a clinical setting that utilizes such results to develop useful prediction and preventive or therapeutic strategies for obesity.

Comparing our performance of predicting overweight and obesity with previous research, our ROC value ∼0.70 is not greater than that of previous research (Mukhopadhyay et al., 2015; Montanez et al., 2017; Ferdowsy et al., 2021; Thamrin et al., 2021). We wish to emphasize that we did not include any anthropometric and clinical phenotypes as predictive features and simply used genome-wide genotype and DNA methylation data in combination with dietary and a few other lifestyle factors without any pre-selection based on prior knowledge. We consider our approach to be an agnostic scan. Additionally, we incorporated interactions with modifiable features in model building, providing insights into developing strategies for the prevention and treatment of obesity.

DNA methylation is an epigenetic process that regulates gene expression without changing the DNA sequence. Genetic factors, modifiable environmental factors (diet and lifestyle), and biological status (considered as an internal environment, such as adiposity status) are believed to influence DNA methylation regulation, which can regulate gene expression and molecular and biological phenotypes. Thus, it is not surprising that 230 DMSs were assessed as important contributors to the best-performing model for predicting obesity, vastly outnumbering the contribution from SNPs at just 21. Notably, this concept can be further supported by our analyses that the functional enrichment was contributed mostly by nearby genes of selected DMSs instead of SNPs. Interestingly, from those selected features, the enriched function in alternative splicing parallels observed correlations between DNA methylation and alternative splicing (Zhang et al., 2020). This enrichment in alternative splicing indicates a potential regulatory mechanism between the genome and environment through DNA methylation (Lev Maor et al., 2015; Gi et al., 2020), possibly acting via recognition of energy intake (Rhoads et al., 2018).

Obesity is a complex disease that is caused by a combination of genetic, biological, socioeconomic, cultural, environmental, and behavioral determinants, and that complexity highlights some of the limitations and challenges in this study. First, we presented one method to integrate different data types in this study, and the development of methods of how to effectively integrate diverse data sets is a focus of ongoing research. Our findings suggest that further investigation is needed in order to integrate multi-omics and modifiable lifestyle factors and to select features to avoid over-fitting from high-dimensional data. Some of those factors are known or potential determinants of obesity, including microbiome data, which were not included in the current research due to a lack of information in our study population. To develop more advanced prediction approaches, a more systematic study design is needed, one that collects data from the individual, environmental, and societal levels. Second, the blood-derived DNA methylation profiles may not be perfectly correlated to expression levels in tissues more relevant to the phenotype under study, such as adipose tissue. Third, although the current work was performed cross-sectionally, this method can be applied to longitudinal data and used to predict the risk of developing obesity. In addition, this methodology can be used in future research to validate our approach of providing personalized nutrition and/or lifestyle recommendations using clinical trials.

In conclusion, we report an integrated approach to predict obesity status using omics and dietary information and ML. Results such as these can inform further development of approaches for prediction models and applying precision nutrition strategies for the prevention and treatment of obesity. We suggest that this current work can be further used to predict other health outcomes and inform modifiable features to improve the status of health and diseases.

## Data Availability

The controlled access datasets were used in this study. This data can be requested and available at dbGaP (https://dbgap.ncbi.nlm.nih.gov) under the accession numbers, phs000007.v25.p9, phs000007.v28.p10, phs000342.v18.p11, and phs000724.v9.p13.
