# Supplementary figures and images for: Using Machine Learning to Predict Obesity Based on Genome-Wide and Epigenome-Wide Gene–Gene and Gene–Diet Interactions
Source: Front Genet. 2022 Jan 3;12:783845. doi: 10.3389/fgene.2021.783845 (PMC8763388; doi:10.3389/fgene.2021.783845)

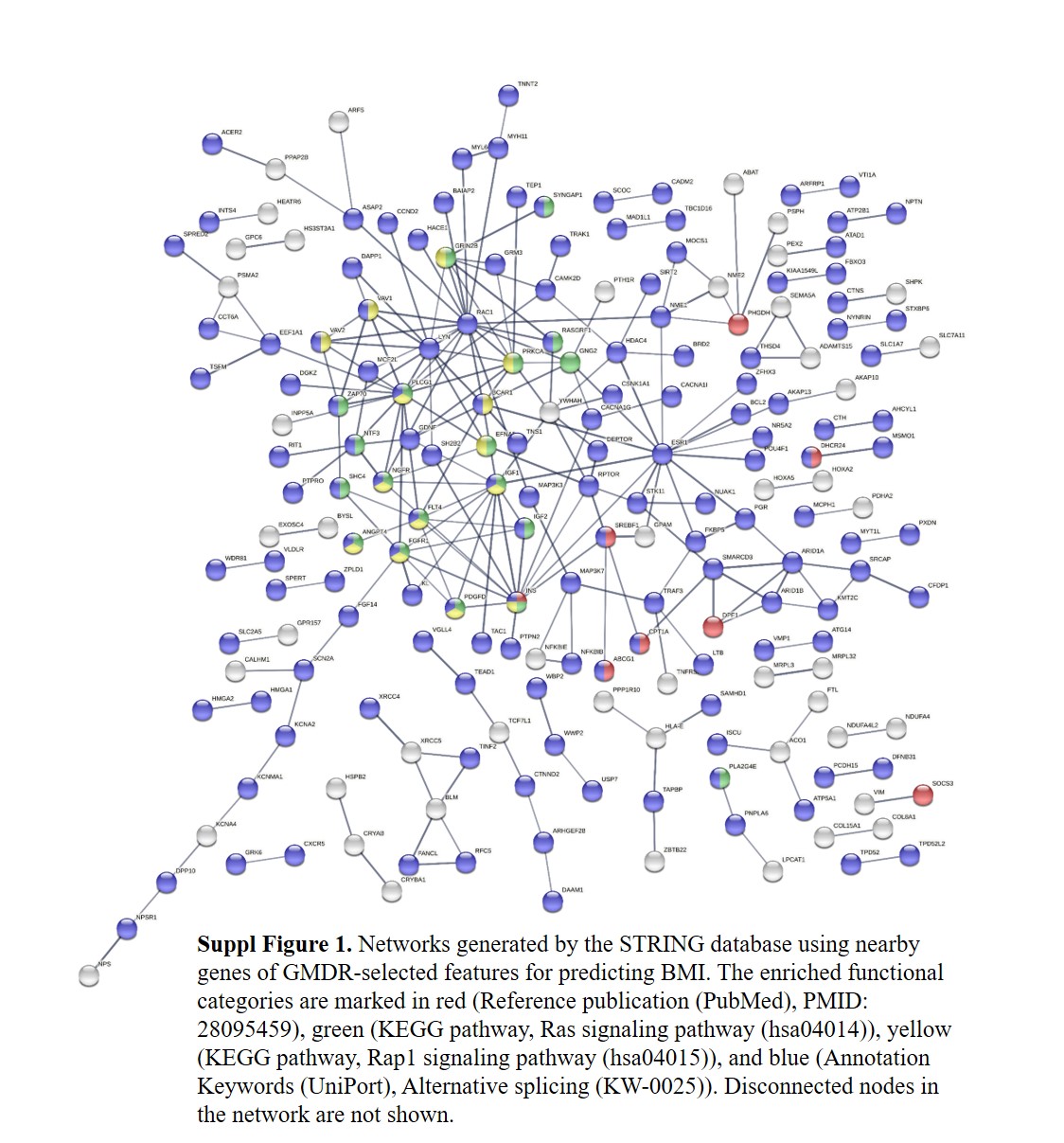

Supplement: Supplementary file 1 [file Image1.JPEG]
